# Surviving Over a Decade With Glioblastoma: A Clinical Course Characterized by Multiple Recurrences, Numerous Salvage Treatments, and Novel Use of Cesium-131 Tiles

**DOI:** 10.7759/cureus.19573

**Published:** 2021-11-14

**Authors:** Shahil Mehta, April K Vassantachart, Croix C Fossum, Wensha Yang, Zhilei L Shen, Ki-Eun Chang, Jason C Ye, Thomas C Chen, Eric L Chang

**Affiliations:** 1 Department of Radiation Oncology, Keck School of Medicine of the University of Southern California, Los Angeles, USA; 2 Department of Neurosurgery, Keck School of Medicine of the University of Southern California, Los Angeles, USA

**Keywords:** stereotactic radio surgery (srs), gammatile, cs 131, surgically targeted radiation therapy, intracranial brachytherapy, radiation oncology, glioblastoma

## Abstract

The prognosis for patients diagnosed with recurrent glioblastoma (GBM) remains poor, with no clear standard of care regarding salvage therapy. Common approaches include chemotherapy, re-resection, tumor treating fields, and reirradiation. However, most studies have shown these to have limited benefits. Reirradiation is particularly difficult due to concern for increased risk of toxicity to surrounding normal brain tissue. A novel intracranial brachytherapy system called GammaTile® (GT Medical Technologies, Tempe, Arizona) involves the placement of Cesium-131 radioactive tiles in the tumor cavity following maximal safe resection. This allows for a highly conformal dose distribution with rapid fall-off to minimize overlap with prior radiation fields and for the application of radiation directly to the high-risk tumor bed. This case report highlights a patient with GBM who survived 11.5 years through multiple recurrences and discusses the many salvage treatments he received, including bevacizumab, irinotecan, and stereotactic radiosurgery (SRS). This case exemplifies that aggressive systemic and local therapies can work well in select patients allowing for long-term survival with a good quality of life. Further efforts should be made to identify which patients may benefit from these therapies. The case study additionally reports on the use of GammaTile therapy. Due to prior external beam radiation therapy and SRS to the treatment site, further external beam radiation options were limited, and the patient was offered GammaTile as local therapy. Although it did not provide a survival benefit in this case due to progressive disease outside of the field of treatment, GammaTile serves as a valuable option in providing local therapy to patients who can no longer receive further radiation. It should be used with careful consideration in lesions characterized by aggressive local invasion.

## Introduction

The prognosis for patients diagnosed with glioblastoma (GBM) remains poor, with a median survival time (MST) of 15-18 months and a five-year overall survival (OS) of 7% with standard therapy [1]. Following initial recurrence, MST is reported to be between 24-44 weeks [1]. Given these poor outcomes and unclear standards of care regarding salvage therapy, the National Comprehensive Cancer Network (NCCN) recommends clinical trial enrollment for eligible patients [2]. Common approaches to treatment include re-resection, nitrosoureas, bevacizumab, re-initiation of temozolomide, and/or tumor treating fields (TTF). Still, studies have shown these salvage options to have limited benefit [1]. Reirradiation is also considered. However, the usefulness is a subject of debate, and there is a concern for increased risk of toxicity to surrounding normal brain tissue [1,2]. In the reirradiation setting, techniques that allow for dose escalation while simultaneously reducing the dose to normal brain tissue may be clinically advantageous.

GammaTile® Therapy (GT Medical Technologies Inc., Tempe, Arizona) is a potential alternative to reirradiation using external beam radiation therapy (EBRT). This novel intracranial brachytherapy system involves the placement of radioactive tiles in the tumor cavity following maximal safe resection. Each bioresorbable collagen tile is 2cm by 2cm and 4mm thick. Each tile has four embedded Cesium (Cs)-131 seeds placed 1cm apart. The decay properties of Cs-131 allow for a rapid dose fall-off beyond the resection cavity, enabling a high degree of conformality that minimizes radiation dose to surrounding tissues. Early clinical data suggest that the procedure is indeed well tolerated with minimal toxicity [3-5].

This case report highlights the remarkably long clinical course of a patient with GBM who survived 11.5 years through multiple recurrences. Along the way, we focus the discussion on the salvage treatments that he received, including bevacizumab and stereotactic radiosurgery (SRS). Additionally, the case study reports on the use of GammaTile therapy. Although it did not prolong survival in the case, we use this as an opportunity to discuss the benefits and limitations of this novel technique.

## Case presentation

A 57-year-old male presented in 2009 with sudden onset aphasia, with a brain MRI demonstrating a 39x34x32mm left parietal cystic mass with ring enhancement. He underwent cyst fenestration with subtotal tumor debulking and was diagnosed with a left parietal GBM, WHO Grade IV. The tumor showed >50% MIB-1 positivity and was positive for GFAP and S-100. MGMT and IDH1 status were not available. Although the tissue was banked and re-reviewed to confirm the diagnosis at a future date, further genetics were never performed on this sample. He received Gamma Knife (GK) SRS to the left parietal target (32x35x33mm) with 16Gy to the 50% isodose line (IDL). He then received intensity-modulated radiation therapy (IMRT) to a dose of 45Gy in 25 fractions (fx) to the left parietal resection bed and residual tumor with a margin with concurrent temozolomide (TMZ), completed three months after surgery. Imaging following his radiation course showed reaccumulating fluid within the cystic component of the mass, and two months later, an Omaya reservoir was placed within the mass to drain fluid as symptoms required. He had clinical and radiographic signs of infection of the Omaya reservoir one month after its placement and had it removed, followed by a course of antibiotics. Unfortunately, he presented again one month later with signs of cranial infection, which required washout and an additional antibiotic course.

He was planned for 6-12 cycles of adjuvant TMZ and had completed two cycles of TMZ before his course of infections. Three months after the Omaya reservoir was removed, when he was deemed clinically stable, he was considered for resumption of his TMZ adjuvant therapy. However, a brain MRI completed at this time showed signs of disease progression. As a result, he was transitioned to bevacizumab and irinotecan combination therapy for 20 months, followed by bevacizumab as a single agent for 15 months, which was stopped due to proteinuria.

His disease remained stable on serial imaging for approximately six years when a brain MRI showed signs of disease progression. He underwent a left posterior parietal subtotal resection four months later, with pathology showing an IDH wildtype astrocytic neoplasm, with some similarities to his prior GBM pathology. MGMT was not evaluated on this specimen. Of note, given the unusual disease course, his original pathology was retrospectively reviewed at this time, and it was confirmed that he did initially have GBM. On multidisciplinary review of the case, this was considered as a recurrence. He later developed hydrocephalus and underwent right frontal ventriculoperitoneal shunt placement shortly after the surgery. He was then restarted on bevacizumab one month later, which was stopped after eight months when a brain MRI showed four new lesions, each under 2cm, suspicious for recurrence. He underwent GK SRS to all four lesions with 15Gy to the 65% IDL to the right occipital lobe, 14Gy to the 50% IDL to the left occipital lobe, and 14Gy to the 50% IDL to two left temporal lobe lesions. This was followed by a cycle of pembrolizumab, which was subsequently stopped due to severe diarrhea, and he continued without systemic therapy. Four months after his prior GK treatment, he was found to have five new lesions in the corpus callosum, pineal region, left temporal lobe, and two lesions in the left occipital lobe, which were each treated with GK SRS with 14Gy to the 50% IDL, 14Gy to the 50% IDL, 14Gy to the 80% IDL and 14Gy to the 60% IDL, respectively.

Use of GammaTile

His follow-up brain MRI, which occurred three months after his last GK treatment, showed a good response of the previously treated lesions as they were overall stable in size. However, when compared to the prior GK treatment planning MRI, there was an increased enhancement in the left temporal lobe anterior to the prior GK treatment location. On multidisciplinary review, this was thought to represent tumor recurrence rather than radiation necrosis. Additionally, he was doing well clinically with no new neurologic deficits on examination and a Karnofsky Performance Status of 90. He was not on systemic therapy, dexamethasone, or anti-epileptics. He was deemed a good surgical candidate and once again was planned for resection of this recurrent lesion with consideration of additional radiotherapy. Given the location of the recurrent disease within the 45Gy volume of the initial IMRT plan and additional dose overlap with multiple prior GK SRS treatments, EBRT options were felt to carry an unacceptable risk of radiation necrosis. As an alternative, the patient was offered resection followed by intracranial brachytherapy with Cesium-131 seeds (GammaTile) implantation. The radiation oncologist reviewed the T1 weighted preoperative MRI with gadolinium to determine the number of tiles needed for the procedure. This was estimated based on the preoperative gross tumor volume and the anticipated cavity volume that would require coverage with 60Gy prescribed to a 5mm depth. Following this assessment, eight GammaTiles containing 32 Cs-131 seeds were preordered before the expected date of surgery. A comprehensive reported protocol, similar to what was completed for this patient, has been published by Ferreira et al [6].

**Figure 1:**
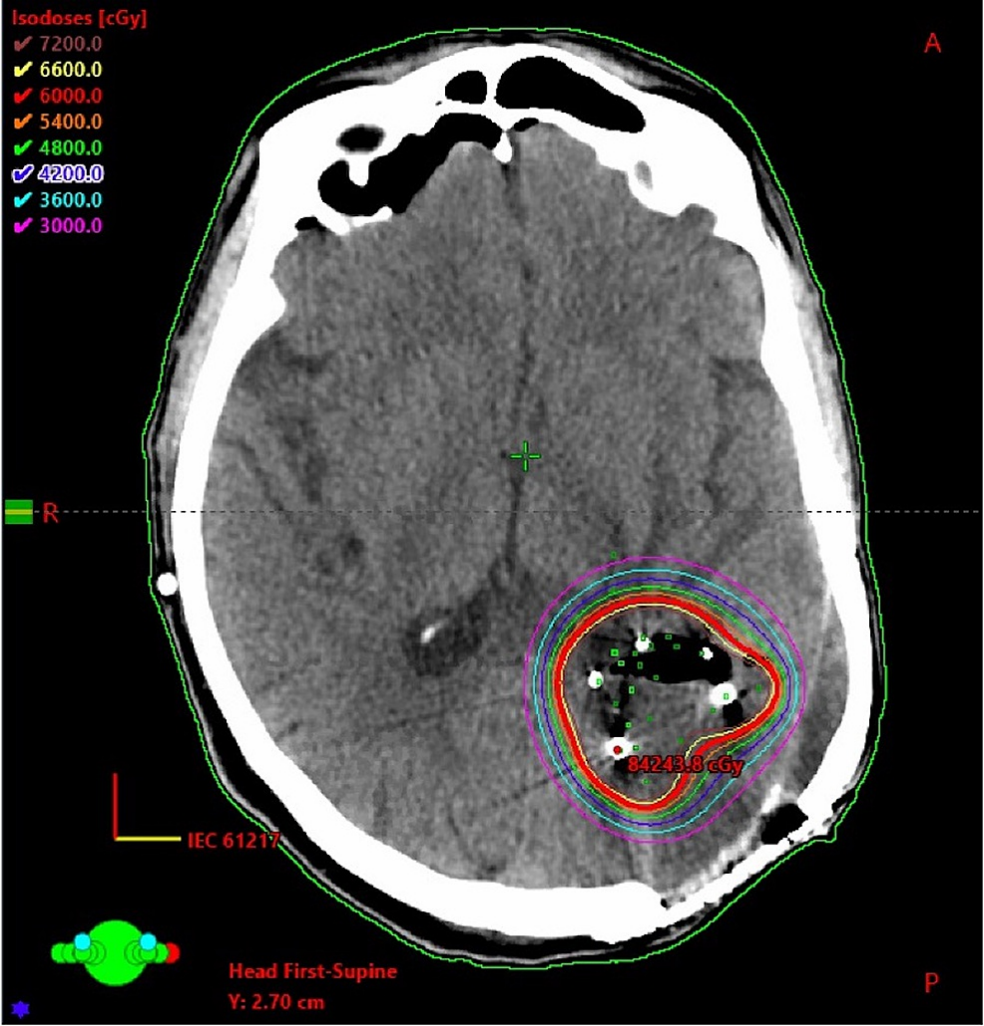
Axial slice of CT Head following Cs-131 GammaTile® placement showing the dose distribution of 60Gy prescribed to a 5mm depth Cs-131: Cesium-131

The patient underwent maximal safe resection and GammaTile placement shortly after this recurrence was discovered. On the day of surgery, numerous tissue samples from various areas of the resection cavity were sent to the frozen section to confirm which areas had tumor relative to radiation necrosis. Maximal safe resection occurred but was limited due to the proximity to the Wernicke’s area and the visual cortex. The surgeon placed a total of eight GammaTiles containing 32 Cs-131 seeds with the assistance of the radiation oncologist. The placement was guided based on the results of the prior biopsies, which identified which areas of the resection cavity had tumor. The placement was evenly spaced with no overlapping GammaTiles in an arrangement sufficient to cover the full extent of the treatment area. The procedure was completed successfully over five minutes. The operative room was surveyed before, during, and after the procedure with no detection of radioactivity above background levels. Post-operative MRI confirmed subtotal resection. Post-operative MRI was then fused with the post-implant CT to determine dose distribution and target coverage. A CT simulation was performed using 1mm slice thickness, and the brachytherapy seeds were delineated by the physicist and confirmed by the radiation oncologist. The gross tumor volume (GTV) was defined as the operative bed adjacent to the placed tiles and expanded 5mm to create a clinical target volume (CTV) to which 60Gy was prescribed to a 5mm depth. The post-implant dosimetry was calculated using the Eclipse™ treatment planning system (Varian Medical Systems Inc., Palo Alto, California). An axial slice from this treatment plan can be seen in Figure 1. The patient was discharged two days after the operation without any complications. Molecular testing was completed on the resected sample following the surgery and showed the tumor to be IDH wild type and MGMT methylated. The final pathology suggested a high-grade astrocytoma. However, in the context of the patient’s prior history and specimens, pathology felt the lesion was compatible with recurrent GBM.

The patient was seen two weeks following the procedure by neurosurgery with subjective complaints of new-onset double vision without any new visual deficits on examination. Shortly after, he presented to the hospital with complaints of nausea, vomiting, aphasia, and weakness. He underwent a CT Head, which was normal, and was placed on dexamethasone 4mg BID, which largely improved his symptoms. One month later, he reported back pain, which prompted an MRI of the spine that revealed diffuse leptomeningeal disease (LMD), most prominently in the thoracic spine. He was treated from T5 to T12 with a dose of 20Gy in five fractions with a three-dimensional conformal radiation therapy plan. Around the same time, he reported worsening of speech and right-sided weakness. A brain MRI showed numerous areas of recurrence and a new mass-like T2 fluid-attenuated inversion recovery (FLAIR) signal abnormality measuring 4.6 x 5.4 x 5.6 cm in the left frontal lobe with extension across the corpus callosum. In the parietal resection cavity where GammaTiles were placed, there was increased enhancement reflecting recurrent disease or inflammatory changes.

A few weeks later, the patient saw neurosurgery and medical oncology and reported worsening aphasia, fatigue, and right-sided weakness. He declined additional treatment and transitioned to comfort care and hospice. He later passed away approximately 11.5 years since his initial GBM diagnosis.

## Discussion

This case report highlights an example of a patient diagnosed with GBM who lived over a decade, likely due to a combination of tumor biology and aggressive therapeutic approaches. Moreover, it is noteworthy that he maintained his functionality and quality of life through multiple recurrences. The timeline of his disease for purposes of discussion can be divided into his initial period of stability following bevacizumab use, his period of aggressive local control through GK SRS, and his GammaTile treatment.

Following his initial recurrence on TMZ, he transitioned to bevacizumab and irinotecan combination therapy for 20 months. This was followed by bevacizumab as a single agent for 15 months. His disease remained stable on serial imaging for approximately six years, which was an unusual result because numerous studies have failed to show a survival benefit from irinotecan or bevacizumab alone or with other therapies [1,2,7,8]. However, it has been demonstrated that bevacizumab is effective at reducing peritumoral edema and other clinical symptoms [9]. In this particular case, bevacizumab appears to have contributed to his improved survival, perhaps due to the specific biology of his tumor and its responsiveness to vascular endothelial growth factor (VEGF) inhibitor therapy, which when applied to all patients does not show a survival benefit [8,10]. Although irinotecan may have contributed to his survival, prior studies do not suggest improved outcomes with this therapy, and current guidelines do not suggest its use. Bevacizumab, however, has weak evidence and is recommended by current guidelines as an option in recurrent GBM [1,2]. Thus, future efforts should evaluate whether select patients may benefit from bevacizumab more than others.

The patient underwent multiple GK SRS sessions for aggressive local control of new lesions later in his treatment course. Reirradiation is controversial for GBM, especially radiosurgery, given the diffuse nature of the disease [2]. A meta-analysis evaluating 50 eligible studies of reirradiation for recurrent GBM reported a six-month OS rate of 73% and a 12-month OS rate of 36%. Although the included studies did not have a comparative cohort and included many studies considered low quality, the pooled results suggested reirradiation may provide local control and possible survival benefits. On subgroup analysis, patients treated with brachytherapy or EBRT in five or fewer fractions appeared to have better outcomes [11].

Regarding the use of SRS specifically, a recent review article compiled published retrospective institutional studies of recurrent GBM treated with SRS. It found the median OS following the initial SRS treatment of a local recurrence to range from 7-13.2 months among these studies [12]. Collectively, the reirradiation data from these publications represent improved outcomes over historical controls. Overall this data is promising but is limited given that it is non-randomized, retrospective, and subject to institutional specific biases and protocols [12]. Nonetheless, efforts should be made to determine whether certain patients, such as the patient reported here, are better candidates for SRS in recurrent GBM.

During his last recurrence, the patient underwent GammaTile placement following maximal safe resection. GammaTile was used rather than EBRT as the patient had previously received prior EBRT and multiple courses of GK SRS to the area. There was a concern for high toxicity if other radiation techniques were used to treat this previously irradiated area. GT technology allows for the treatment of a rim of tissue around the resection cavity to a high dose with a rapid fall-off into the surrounding brain parenchyma, which provides an opportunity for local control with reduced risk of radionecrosis. Overall, this leads to very conformal and localized treatment plans. Previous studies evaluated the use of these implanted Cs-131 seeds in brain metastases, meningiomas, and gliomas and showed a favorable toxicity profile [4,5,13-15]. Additional benefits of this technique include immediate post-resection initiation of radiation in a rapidly proliferating tumor and the relative ease of use when applying the tiles [3].

Published data on GammaTile use for GBM is limited. Abstract data on GammaTile use for previously irradiated GBMs included 28 patients with locally recurrent GBMs. The median OS was 10.7 months and radiographic local recurrence was 8.8 months [5]. Due to the patient’s rapid progression and development of LMD, the extent and benefit of local control of this patient’s resection cavity are unclear. GammaTile likely did not provide him survival benefit but it is possible his GammaTile treatment delayed the progression of symptoms by slowing down tumor growth in the resection cavity. However, GammaTile may have also caused his visual complaints two to three weeks following treatment, although his disease progression may have also caused this. An additional benefit to note is that he did not require other visits to the radiation oncology department to deliver additional treatment as would have been required with EBRT. This novel technology should be evaluated further to help determine its role in treating aggressive intracranial malignancies.

## Conclusions

Recurrent glioblastoma has historically been challenging to manage with no standard of care treatment. Despite the often poor prognosis for these patients, this case report exemplifies that aggressive systemic and local therapies can work well in select patients allowing for long-term survival with a good quality of life. His case shows that treatments still considered investigational for recurrent GBM, such as bevacizumab and SRS, may benefit select patients. Further efforts should be made to determine which patients may benefit from these therapies. Additionally, Cs-131 GammaTiles may be a valuable tool for local therapy in cases where reirradiation with EBRT is not possible. They should be further evaluated to determine which clinical scenarios are best suited for treatment with them.
